# Long noncoding RNA NORAD, a novel competing endogenous RNA, enhances the hypoxia-induced epithelial-mesenchymal transition to promote metastasis in pancreatic cancer

**DOI:** 10.1186/s12943-017-0738-0

**Published:** 2017-11-09

**Authors:** Hongzhe Li, Xinjing Wang, Chenlei Wen, Zhen Huo, Weishen Wang, Qian Zhan, Dongfeng Cheng, Hao Chen, Xiaxing Deng, Chenghong Peng, Baiyong Shen

**Affiliations:** 1 Research Institute of Pancreatic Disease, Ruijin Hospital, School of Medicine, Shanghai Jiao Tong University, Shanghai, China; 2 Pancreatic Disease Centre, Ruijin Hospital, School of Medicine, Shanghai Jiao Tong University, Shanghai, China; 3 Shanghai Institute of Digestive Surgery, Ruijin Hospital, School of Medicine, Shanghai Jiao Tong University, Shanghai, China

## Abstract

**Background:**

Pancreatic cancer, one of the top two most fatal cancers, is characterized by a desmoplastic reaction that creates a dense microenvironment, promoting hypoxia and inducing the epithelial-to-mesenchymal transition (EMT) to facilitate invasion and metastasis. Recent evidence indicates that the long noncoding RNA NORAD may be a potential oncogenic gene and that this lncRNA is significantly upregulated during hypoxia. However, the overall biological role and clinical significance of NORAD remains largely unknown.

**Methods:**

NORAD expression was measured in 33 paired cancerous and noncancerous tissue samples by real-time PCR. The effects of NORAD on pancreatic cancer cells were studied by overexpression and knockdown in vitro. Insights into the mechanism of competitive endogenous RNAs (ceRNAs) were gained from bioinformatics analyses and luciferase assays. In vivo, metastatic potential was identified using an orthotopic model of PDAC and quantified using bioluminescent signals. Alterations in RhoA expression and EMT levels were identified and verified by immunohistochemistry and Western blotting.

**Results:**

NORAD is highly expressed in pancreatic cancer tissues and upregulated in hypoxic conditions. NORAD upregulation is correlated with shorter overall survival in pancreatic cancer patients. Furthermore, NORAD overexpression promoted the migration and invasion of pancreatic carcinoma cells, while NORAD depletion inhibited EMT and metastasis in vitro and in vivo. In particular, NORAD may function as a ceRNA to regulate the expression of the small GTP binding protein RhoA through competition for hsa-miR-125a-3p, thereby promoting EMT.

**Conclusions:**

Elevated expression of NORAD in pancreatic cancer tissues is linked to poor prognosis and may confer a malignant phenotype upon tumor cells. NORAD may function as a ceRNA to regulate the expression of the small GTP binding protein RhoA through competition for hsa-miR-125a-3p. This finding may contribute to a better understanding of the role played by lncRNAs in hypoxia-induced EMT and provide a potential novel diagnostic and therapeutic target for pancreatic cancer.

**Electronic supplementary material:**

The online version of this article (10.1186/s12943-017-0738-0) contains supplementary material, which is available to authorized users.

## Background

In 2012, an estimated 338 000 new cases of pancreatic cancer were diagnosed worldwide, while the disease was responsible for 331 000 deaths [1]. In contrast to the steady increase in survival rates for most cancers, advances have been slow for pancreatic cancers, for which the 5-year relative survival remained at approximately 8% for 2005–2011, only 4–5% higher than 1975–1989 [2]. Both morbidity and mortality continue to increase. Pancreatic cancer is projected to be one of the top two cancers in terms of fatalities in the next ten years [2, 3]. At present, surgical resection offers the only chance for a cure. Unfortunately, 80–85% of patients present with advanced metastasis and unresectable tumors when diagnosed [4]. Metastasis is the leading cause of death among pancreatic cancer patients [5]. However, the underlying molecular mechanisms that trigger metastasis remain unclear. It is believed that the hypoxic microenvironment and hypoxia-induced epithelial-to-mesenchymal transition (EMT) are critical drivers of pancreatic cancer metastasis and progression [6–8]. These processes occur via activation of various signaling pathways such as Hedgehog signaling [7, 9], PI3K/Akt signaling [10] and Notch signaling [11].

Recent studies have revealed that the aberrant expression of noncoding RNAs plays an important role in gene control and may play a role in cancer biology as oncogenes or tumor suppressor genes [12]. Existing evidence shows that several noncoding RNAs, such as miR-1236 [13], miR-30c [14] and miRNA-34a [15], also participate in regulating hypoxia-induced EMT. However, it remains to be shown whether long noncoding RNAs (lncRNAs) participate in modulating hypoxia-induced EMT.

It has been reported that lncRNAs can act as competing endogenous RNAs (ceRNAs) or “RNA sponges”, interacting with microRNAs in a manner that can sequester these molecules and reduce their regulatory effect on target mRNAs [16, 17]. In other words, lncRNAs, mRNA transcripts and false gene transcripts can affect each other by competitively combining with a miRNA response element (MRE) to influence post-transcriptional regulation [18].

For instance, the long non-coding RNA Unigene56159 promotes metastasis by acting as a ceRNA for miR-140-5p in hepatocellular carcinoma cells [19]. The lncRNA HOTAIR may act as a ceRNA, effectively becoming a sink for miR-331-3p, thereby modulating the derepression of HER2 and promoting the proliferation, migration and invasion of gastric carcinoma cells [20].

NORAD (annotated as LINC00657 in RefSeq) is a highly abundant, conserved mammalian noncoding RNA that can directly regulate both ploidy and chromosomal stability by sequestering PUMILIO proteins [21]. Furthermore, NORAD is reported to be a potential oncogenic gene and is associated with overall survival in breast cancer [22]. NORAD is more highly expressed in the cytoplasm and is significantly upregulated by hypoxia in endothelial cells [23]. However, the mechanisms behind these events have not been revealed.

In the present study, we demonstrated that the lncRNA NORAD is upregulated during hypoxia and promotes EMT in tumor cells. NORAD facilitates invasion and metastasis by increasing the expression of the small GTP binding protein RhoA in vitro and in vivo. Mechanistically, NORAD may function as a ceRNA to regulate the expression of RhoA through competition for miR-125a-3p, thus playing an oncogenic role in the pathogenesis of pancreatic cancer. We also found that NORAD is upregulated in pancreatic ductal adenocarcinomas (PDACs). Elevated NORAD expression in tumor tissues is linked to poor prognosis and recurrence, demonstrating the clinical significance of this molecule during cancer progression.

## Methods

### Microarray analysis and bioinformatics methods

Expression profiles were obtained from the GEO database (www.ncbi.nim.nih.gov/geo/) as mentioned previously [24]. Expression data from 55 pancreatic tumor tissues and their matched non-tumor tissues from two human microarray datasets GSE15471 [25] and GSE16515 [26] (39 pairs from GSE15471 and 16 pairs from GSE16515) were obtained and compared using the robust multiarray average (RMA) algorithm. Next, we used Venn analysis to analyze aberrant lncRNAs. Afterward, GSE32688 [27] was analyzed for the expression of miR-125a-3p and RhoA. In addition, box plots (Additional file 1: Figure S1) were made to graphically view the value distributions among datasets. By analyzing the boxplots, these databases were confirmed to be median-centered and cross-comparable.

The potential microRNA binding sites within NORAD were predicted by computer-aided algorithms obtained from Starbase v2.0 (http://starbase.sysu.edu.cn) [28, 29]. MicroRNA-mRNA binding sites were obtained from miRTarBase (http://mirtarbase.mbc.nctu.edu.tw) [30].

### Clinical specimens

PDAC specimens and the corresponding adjacent non-tumor tissues were obtained from patients who had undergone curative surgery at Shanghai Jiao Tong University School of Medicine Affiliated Ruijin Hospital between 2013 and 2015. None of them had received radiotherapy or chemotherapy before their surgery. Staging had been performed clinically, radiographically, and pathologically. The fresh primary PDAC tissues and adjacent non-tumor pancreas tissue samples were collected and snap-frozen in liquid nitrogen immediately after resection, then stored at −80 °C for further use.

### Cell culture and hypoxia model

HEK-293Ts and the pancreatic cancer cell lines SW1990, Capan-1, PANC-1, AsPC-1, CFPAC-1, MIAPaCa-2, and BxPC-3 were purchased from ATCC (www.atcc.org). Premalignant HPDE cells (HPV16-immortalized normal pancreatic ductal epithelium) were generously provided by Shanghai Cell Bank of the Chinese Academy of Sciences. All cell lines were tested and authenticated by DNA typing at the Shanghai Jiao Tong University Analysis Core. All cells were cultured in DMEM, IMDM or RPMI 1640 (Gibco) with 10% Fetal Bovine Serum (FBS, Gibco) at 37 °C under 5% CO2 in a humidified chamber.

During hypoxic treatment conditions [31], cells were cultured in a modular incubator chamber (Billups-Rothenberger) that was flushed with 1% O_2_, 5% CO_2_, and 94% N_2_. Cells were harvested after 48 h. During normoxic control treatments, cells were cultured in an incubator with a humidified atmosphere containing 5% CO_2_ and 20% O_2_.

### Quantitative real-time PCR

Total RNA was isolated from cells with TRIzol reagent (Invitrogen, Carlsbad, CA, USA). Next, cDNA was synthesized with a reverse transcription kit (Invitrogen, Carlsbad, CA, USA) according to the manufacturer’s instructions. qRT-PCR was performed on the 7500 Fast Real-time PCR system (Applied Biosystems) using a standard protocol from the SYBR Green PCR Kit (Toyobo). The results were normalized to the expression of GAPDH. Primers used for qRT-PCR assays are listed in Additional file 2: Table S1. For the detection of miRNA expression, reverse transcription was performed and microRNAs were detected with stem-loop primers purchased from RiboBio (Guangzhou, China). U6 snoRNA was used as the endogenous control. Relative fold changes were calculated using the 2^-△△Ct^ method. All PCR assays were repeated three times.

### Plasmid constructs

NORAD cDNA was cloned into the mammalian expression vector pcDNA3.1 (Invitrogen). NORAD cDNA fragments containing either the predicted potential microRNA binding sites (wildtype, wt) or scrambled microRNA binding site sequences (mutation, mut) were amplified by PCR. Moreover, plasmids containing the firefly luciferase reporter were constructed with RhoA-3′-UTR-wildtype ( RhoA-3′-UTR-wt-luc) and RhoA-3′-UTR-mutation (mutated miR-125a-3p binding region; RhoA-3′-UTR-mut-luc). Mimics and inhibitors of hsa-miR-125a-3p and rno-miR-239b-5p were purchased from RiboBio (Guangzhou, China). rno-miR-239b-5p was used as a negative control.

### Virus packaging and infection

shRNA sequences targeting NORAD were designed, inserted in lentiviral plasmids and then transfected into HEK-293Ts with virus packaging plasmids to produce the lentivirus. The viral supernatants were collected at 48 and 72 h after transfection and added into PANC-1 and SW1990 cells to construct the stably transfected cell lines. Puromycin was added to the media 48 h after infection and maintained for at least 1 week to select stably transfected cell lines (PANC-1/SW1990-sh-NORAD or PANC-1/SW1990-sh-NC).

### Wound healing assay

PDAC cells (1 × 10^6^ cells/well) were treated with different reagents, seeded in six-well plates and cultured until they reached confluence. Wounds were made in the cell monolayer by making a scratch with a 20 μL pipette tip. Plates were washed once with fresh medium after 24 h in culture to remove non-adherent cells. Following this wash, plates were photographed.

### Transwell migration and invasion assay

Approximately 1 × 10^5^ PDAC cells treated with different reagents were suspended in serum-free medium and seeded in either Matrigel-coated chambers with 8-μm pores (BD Biosciences, Franklin Lakes, NY, USA) for migration assays or in chambers (Corning Costar, NY, USA) that were not coated with Matrigel for invasion assays. For both assays, medium containing 10% FBS was added to the lower chamber as a chemoattractant. After 12 h, the cells that had invaded through the membrane to the lower surface were fixed with methanol, stained with 0.5% crystal violet solution and counted in 5 random fields of view (200×). RhoA pathway specific inhibitor CCG-1423 [32] was purchased from selleck and used as recommended.

### In vivo orthotopic model

Athymic BALB/C mice (male, 4–6 weeks old) were purchased from the Chinese Academy of Sciences (Shanghai, China) and were maintained in a specific pathogen-free facility. Orthotopic implantation of pancreatic cancer cell lines was performed as described previously [33]. For experimental metastasis assays, the SW1990-sh-NORAD and SW1990-sh-NC cell lines were labeled with firefly luciferase. Each mouse was then injected with 100 μL of cell suspension (1 × 10^6^ cells) into the pancreas. Animal health conditions and metastatic progression were monitored. Metastasis was quantified using a noninvasive bioluminescence In Vivo Imaging System (IVIS, Xenogen) as described previously [34]. All mice were sacrificed and both the primary tumor and metastatic foci were observed by general observation, H & E staining and immunohistochemical staining.

### Dual-luciferase reporter assay

Recombinant plasmids or an empty plasmid encoding the firefly luciferase reporter were transiently transfected into SW1990-sh-NORAD and SW1990-sh-NC cells using Lipofectamine 2000 (Invitrogen, USA) The renilla luciferase reporter pRL-CMV plasmid (Promega) was also transfected into these cells as an internal control. Each group above was transfected with miR-125a-3p mimic or a negative control. Luciferase activities in the indicated cells were measured using the Dual-Luciferase Reporter Assay System (Promega, USA) 48 h after transfection according to the manufacturer’s instructions.

### Western blot assay

Cells were lysed with RIPA cell lysis buffer in the presence of a protease inhibitor cocktail (Sigma, USA) in order to harvest total cell protein. Standard Western blotting was carried out as described previously [35]. In brief, the BCA Protein Assay Kit (Pierce, USA) was used to quantify the protein concentration of each cell lysate. Equal amounts of protein samples were loaded onto a 10% SDS-PAGE gel and then transferred onto PVDF membranes. After blocking with skim milk, the membranes were incubated with primary antibodies diluted in TBST buffer overnight at 4 °C and then with the HRP-conjugated secondary antibody for 2–3 h at room temperature. Finally, the images of protein bands were captured by a Tanon detection system using ECL reagent (Thermo).

### Statistical analysis

Data were presented as the mean ± one standard deviation (SD) from at least three separate experiments. The Student t test, X^2^ test, log-rank test, nonparametric Mann–Whitney U test, or Fisher exact test was used for comparisons between groups. The Kaplan–Meier method was used to estimate overall survival (os) and recurrence rate. Differences were deemed statistically significant at *P* < 0.05. The results are shown as the mean ± SEM.

### Study approval

This study was approved by the Clinical Research Ethics Committee of Shanghai Jiao-Tong University. All patients were fully informed of the experimental procedures before tissue acquisition. All animal experimental procedures were performed in compliance with the institutional ethical requirements and were approved by the Shanghai Jiao-Tong University School of Medicine Committee for the Use and Care of Animals.

## Results

### LncRNA expression profiles in PDAC

**Fig. 1:**
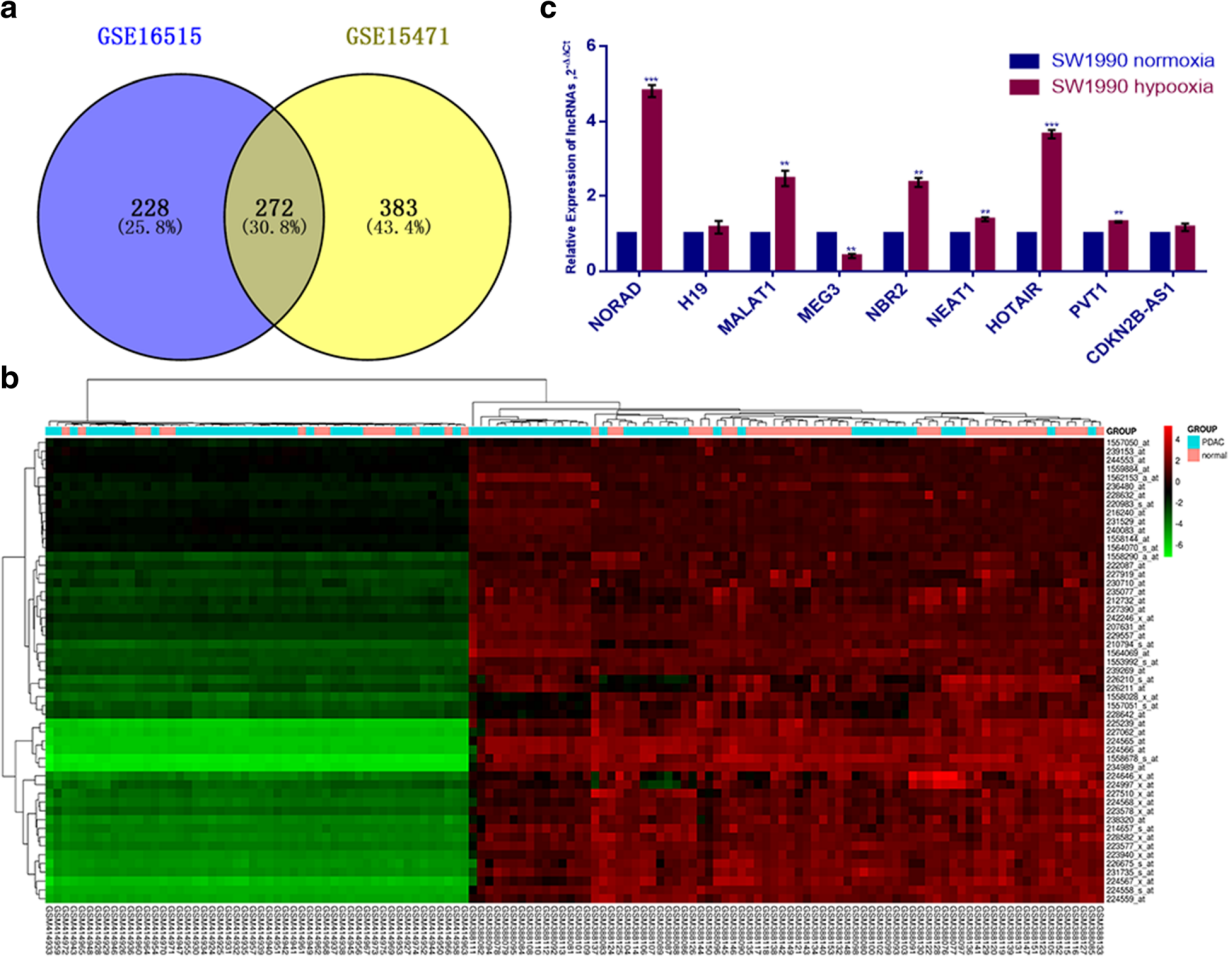
Analysis of lncRNA expression profiles in PDAC databases indicates that NORAD increases during hypoxia. **a** Venn analysis of non-coding RNAs that are significantly upregulated or downregulated in GSE15471 and GSE16515; **b** Heatmap of hypoxia-related lncRNA expression profiles; **c** Relative expression of hypoxia-related lncRNAs in SW1990 after 48 h of hypoxic or normoxic treatment. *, statistical significance, *P* < 0.05, **p < 0.01, ***p < 0.001

To identify eligible lncRNAs in PDAC, we analyzed microarray expression profiles. Two human PDAC microarray datasets (GSE15471 and GSE16515) were selected and analyzed for consistently aberrant lncRNAs between PDAC and normal pancreas tissues using GEO2R. Fold changes in robust multiarray average (RMA) signal intensities (which represent expression differences between tumor and normal tissue) were calculated and cutoff values were set at |RMA fold change| > 1.5 and a *p* value < 0.05. Subsequently, we identified 272 consistent non-coding RNAs that were significantly upregulated or downregulated by Venn analysis (Fig. 1a).

### NORAD was notably increased during hypoxia

As is already known, intratumoral hypoxia plays a crucial role in pancreatic cancer metastasis. After the above analyses, aberrant lncRNAs (Fig. 1b) with potential roles in the hypoxia pathway were chosen for further study.

To evaluate hypoxia-induced alterations in lncRNA expression, the pancreatic cancer cell line SW1990 was cultured under hypoxic or normoxic conditions for 48 h. We found that NORAD was significantly increased after hypoxic stimulation (Fig. 1c). Additionally, we found that NEAT1, PVT1, NBR2, HOTAIR, MALAT1 and NORAD increased during hypoxia, while MEG3 decreased during hypoxia.

### NORAD promotes tumor metastasis in vitro and in vivo

As NORAD was upregulated under hypoxia-stimulating conditions, we hypothesized that superfluous NORAD may participate in the development of hypoxia-induced malignant phenotypes.

**Fig. 2:**
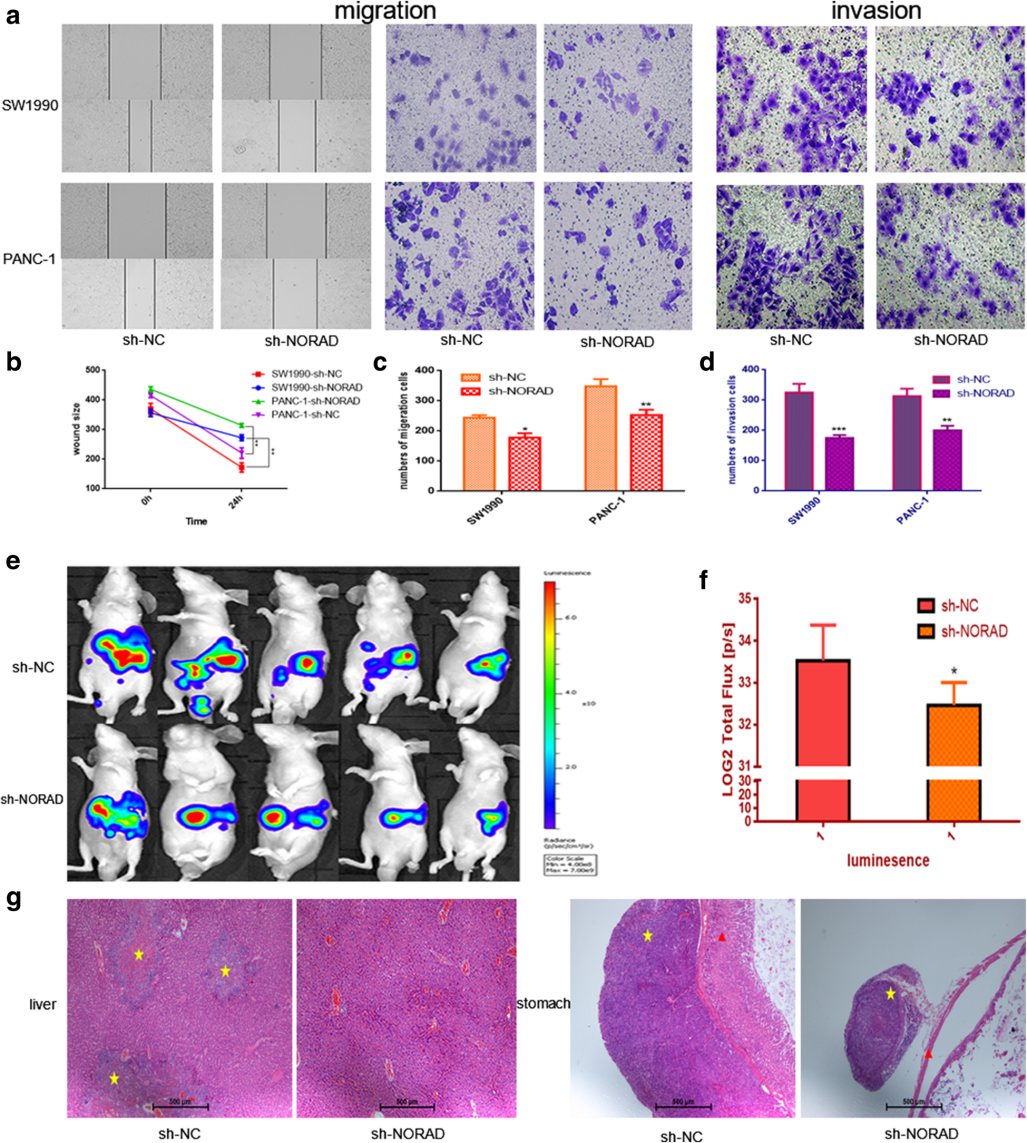
NORAD promotes cell migration and invasion in vitro and in vivo. **a** The migration and invasion abilities of tumor cells were assessed using wound healing assays as well as transwell migration and invasion assays. Left panel: wound healing assays (photographed after 24 h), middle panel: transwell migration assays (photographed after 12 h), right panel: transwell invasion assays (photographed after 12 h); **b** The distances between wound edges after 0 h and 24 h; **c**-**d** Histograms showing the numbers of cells that have migrated or invaded after 0 h and 12 h; **e** SW1990-sh-NORAD and SW1990-sh-NC were labeled with firefly luciferase and injected into the abdominal cavity of nude mice (*n* = 5). The bioluminescent signal was assessed 3 weeks after injection; **f** Histogram showing the bioluminescent signal intensity detected using a noninvasive In Vivo Imaging System; **g** Representative H & E staining images of liver and gastric wall from the different groups. Yellow star: micrometastatic folic, red triangle: gastric mucosa. *p < 0.05 **p < 0.01, ***p < 0.001

To investigate further, we selected SW1990 and PANC-1, two pancreatic cell lines with highest NORAD expression among 8 cell lines (shown in Additional file 3: Figure S2), and knocked down NORAD using shRNAs and then assessed tumor cell migration and invasion abilities using wound-healing assays as well as transwell migration and invasion assays. As shown in Fig. 2a-d, knockdown of NORAD in PANC-1 or SW1990 cells markedly suppressed cell migration and invasion compared with their respective control cells.

To further quantify metastatic and dissemination potential in vivo, we established an orthotopic metastatic model, as mentioned above. SW1990-sh-NORAD and SW1990-sh-NC cells were labeled with firefly luciferase and injected into the pancreas of nude mice. After 3 weeks, the scope of metastasis was quantified by non-invasively detecting the bioluminescent signal on a bioluminescence In Vivo Imaging System (Fig. 2e-f). Signals from the group injected with SW1990-sh-NC cells were significantly stronger than those from the group injected with SW1990-sh-NORAD cells (*p* = 0.043). Moreover, more distant micrometastatic foci were observed in the negative control group than the sh-NORAD group by H & E staining. It is very obvious that metastasis was repressed when knocking down NORAD (Fig. 2g).

### NORAD inhibits miR-125a-3p via direct binding

**Fig. 3:**
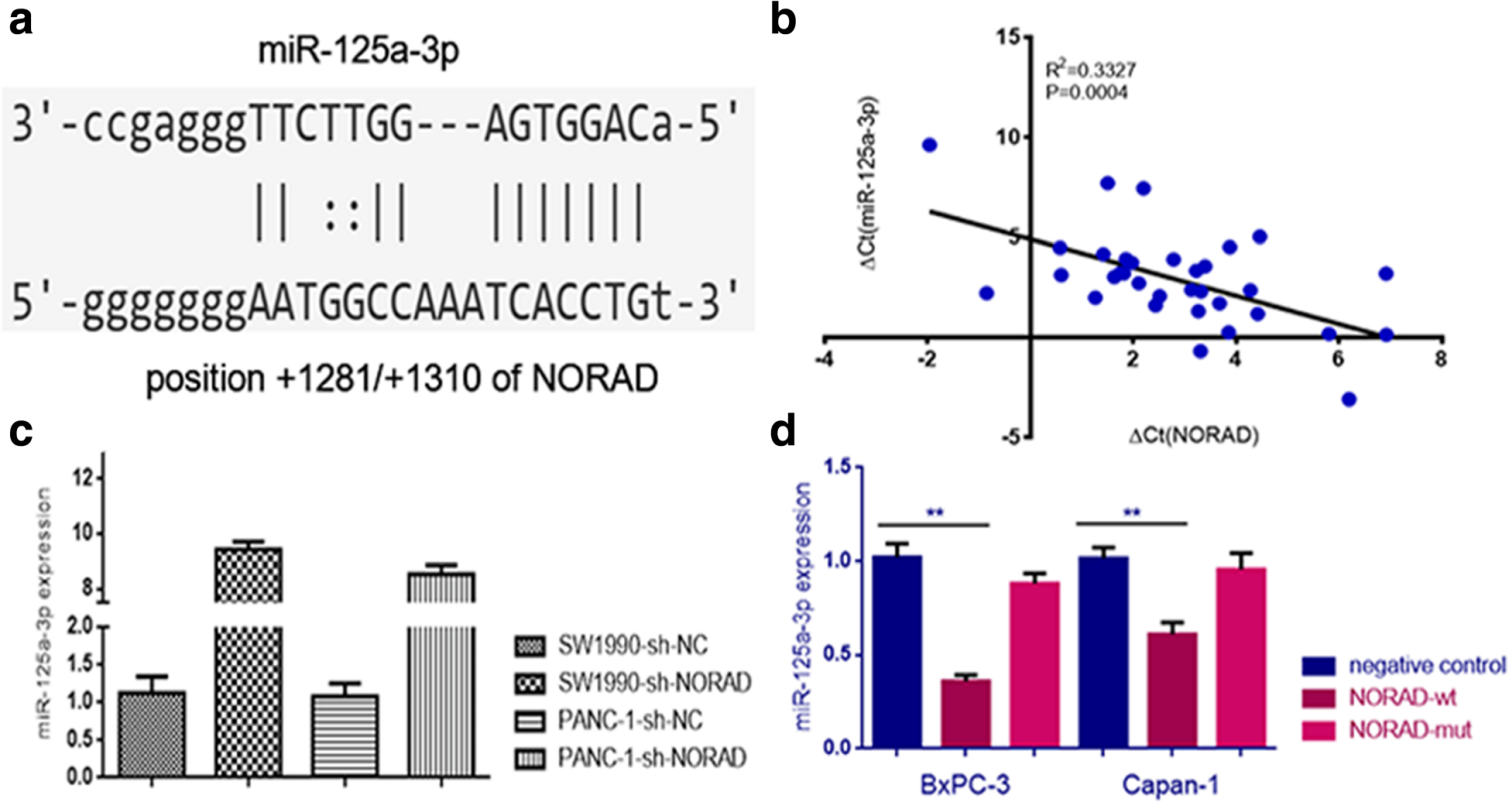
NORAD can directly bind to miR-125a-3p and inhibit its expression. **a** NORAD contains a sequence that is complementary to miR-125a-3p; **b** the expression of NORAD was negatively associated with the expression of miR-125a-3p in 33 PDAC tissues; **c** miR-125a-3p was upregulated when NORAD was knocked down in SW1990 and PANC-1; **d** Relative expression of miR-125a-3p was significantly decreased when NORAD was overexpressed in BxPC-3 and Canpan-1, whereas this phenomenon disappeared when the NORAD MRE was mutated. *, statistical significance, *P* < 0.05, **p < 0.01, ***p < 0.001

Accumulating evidence has suggested that lncRNAs might function as ceRNAs by binding to miRNAs and functionally liberating other RNA transcripts [17]. To examine whether NORAD operates by a similar mechanism in PDAC cells, we used Starbase v2.0 to predict the potential miRNA binding sites in NORAD and found a sequence complementary to miRNA-125a-3p (Fig. 3a). Furthermore, we found that the expression of NORAD was negatively associated with the expression of miR-125a-3p in 33 PDAC tissues (Fig. 3b). We then tested the effects of shRNA-mediated downregulation of NORAD on the expression of miR-125a-3p. Notably, miR-125a-3p was upregulated in the sh-NORAD-treated SW1990 and PANC-1 cells compared with negative control-treated cells (Fig. 3c). Moreover, we selected BxPC-3 and Canpan-1, two pancreatic cell lines with lowest NORAD expression among 8 cell lines (shown in Additional file 3: Figue S2), and overexpress NORAD with plasmids containing wildtype NORAD (NORAD-wt) or NORAD with mutation in its MRE (NORAD-mut) respectively. After we overexpressed NORAD in BxPC-3 and Canpan-1, we found a significant decrease in miR-125a-3p. This phenomenon disappeared when the MRE of NORAD was mutated (Fig. 3d), suggesting that the lncRNA NORAD can repress the expression of miR-125a-3p via direct binding at the MRE.

### NORAD increases the expression of RhoA acting as a ceRNA of miR-125a-3p

**Fig. 4:**
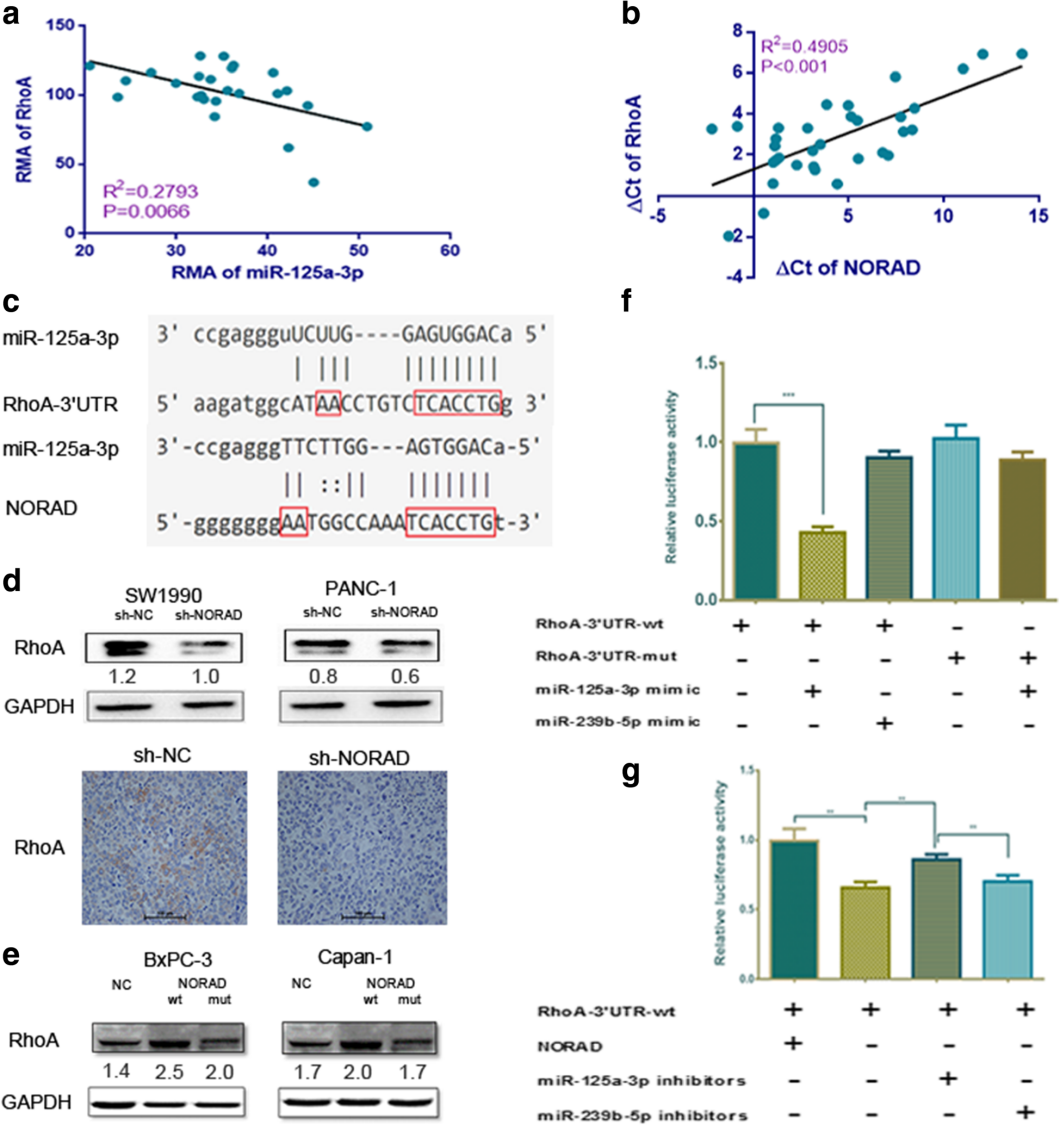
NORAD increases the expression of RhoA and acts as a ceRNA for miR-125a-3p. **a** RhoA expression is negatively correlated with miR-125a-3p in GSE32688. The results were expressed as RMA Values; **b** NORAD and RhoA mRNA levels are positively associated in 33 PDAC tissues; **c** miR-125a-3p can complement the seed region of RhoA, which is similar to the NORAD MRE. **d** NORAD increases RhoA protein level. Upper panel: western blot of RhoA was performed. Relative density was shown below each lane. Bottom panel: IHC staining of orthotopic implantation tumor tissues; **e** RhoA protein level is increased by NORAD-wt but not by NORAD-mut; **f** The 3′-UTR of RhoA (wt or mut) was fused to the luciferase coding region and transfected into SW1990 with along with a miR-125a-3p mimic to confirm that RhoA is a target of miR-125a-3p. A miR-239b-5p mimic was used as a negative control; **g** RhoA-3′-UTR-wt-luc was transfected into SW1990-sh-NC or SW1990-sh-NORAD. Inhibitors of miR-125a-3p or miR-239b-5p were added. Rno-miR-239b-5p was used as a negative control. The histogram indicates the luciferase values measured 48 h after transfection. *, statistical significance, *P* < 0.05, **p < 0.01, ***p < 0.001

Using miRTarBase, we found that RhoA was a potential target of miR-125a-3p. Additionally, we found that RhoA expression is negatively correlated with miR-125a-3p in GSE32688 [27] (data provided in Additional file 4: Table S2) and that miR-125a-3p can complement the seed region of RhoA. Interestingly, the MRE of NORAD was found to be similar to the seed region of RhoA and the expression levels of NORAD and RhoA were found to be positively correlated (Fig. 4a-c). RhoA is downregulated after NORAD knockdown in vitro and vivo (Fig. 4d). And we also found that RhoA is increased by NORAD-wt, while NORAD-mut failed to increase RhoA (Fig. 4e). This suggests that NORAD may enhance RhoA by repressing miR-125a-3p. To confirm the binding in predicted sites of RhoA-3′-UTR, we mutated binding sites in RhoA and then performed reporter assays. We found that miR-125a-3p could inhibit the activity of RhoA-3′-UTR-wt, but had no influence on the activity of RhoA-3′-UTR-mut (Fig. 4f). In addition, we found that downregulation of NORAD could significantly inhibit the activity of RhoA-3′-UTR. This effect could be partially retrieved using a miR-125a-3p inhibitor (rather than miR-239b-5p), which suggests that NORAD competes with the RhoA-3′-UTR for miR-125a-3p (Fig. 4g).

### NORAD promotes EMT via regulating RhoA expression

**Fig. 5:**
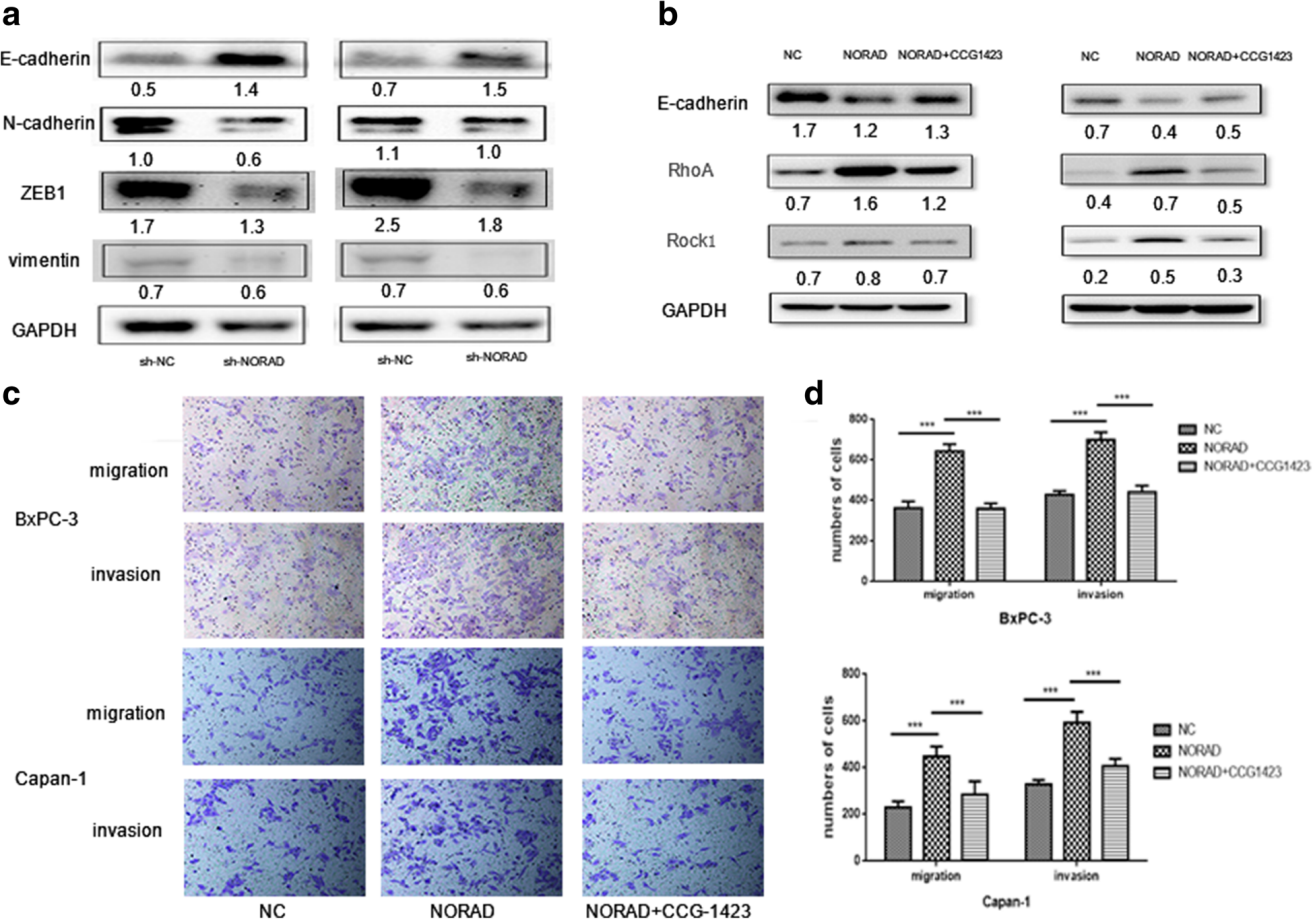
NORAD promotes EMT via regulating RhoA expression. **a** Western blot analysis of E-cadherin, N-cadherin, Vimentin and ZEB1. GAPDH was used as a loading control. Relative density was shown below each lane; **b** Western blot analysis of E-cadherin, RhoA and RhoA downstream protein Rock1. Relative density was shown below each lane; **c** transwell migration and invasion assays, NC: negative control, NORAD: cell lines with NORAD overexpressed, NORAD + CCG-1423: NORAD overexpressed cell lines incubated with 300 nM CCG-1423 72 h; **d** Histograms of numbers of migration and invasion cells*, statistical significance, *P* < 0.05, **p < 0.01, ***p < 0.001

As we know, hypoxic stimuli can trigger the expression of RhoA and its downstream effector ROCK1, leading to cell metastasis, and RhoA plays a crucial role in mediating the epithelial-mesenchymal transition (EMT) [36]. To further investigate the molecular mechanisms underlying these events, we explored whether NORAD regulates EMT in cancer cells. As shown in Fig. 5a-b, knockdown of NORAD significantly decreased the expression levels of the mesenchymal cell markers N-cadherin, Vimentin, and ZEB1 but increased the expression levels of the epithelial cell marker E-cadherin. And overexpression of NORAD resulted in RhoA increasing and E-cadherin decreasing. This suggests that the more NORAD expressed the more RhoA protein will be produced, and then triggering more aggressive EMT and cell migration and invasion. Furthermore, as Fig. 5b-d shown, when we treated cell lines with RhoA pathway specific inhibitor CCG-1423 [32], RhoA downstream protein ROCK1 decreased and aggressive EMT and invasive behaviors caused by NORAD overexpression were partly redressed. In another words, blocking RhoA activity with the RhoA inhibitor can impede the flow of EMT induced by NORAD, indicating that NORAD promotes EMT via regulating RhoA.

### Excess NORAD is associated with poor prognosis in PDAC

**Fig. 6:**
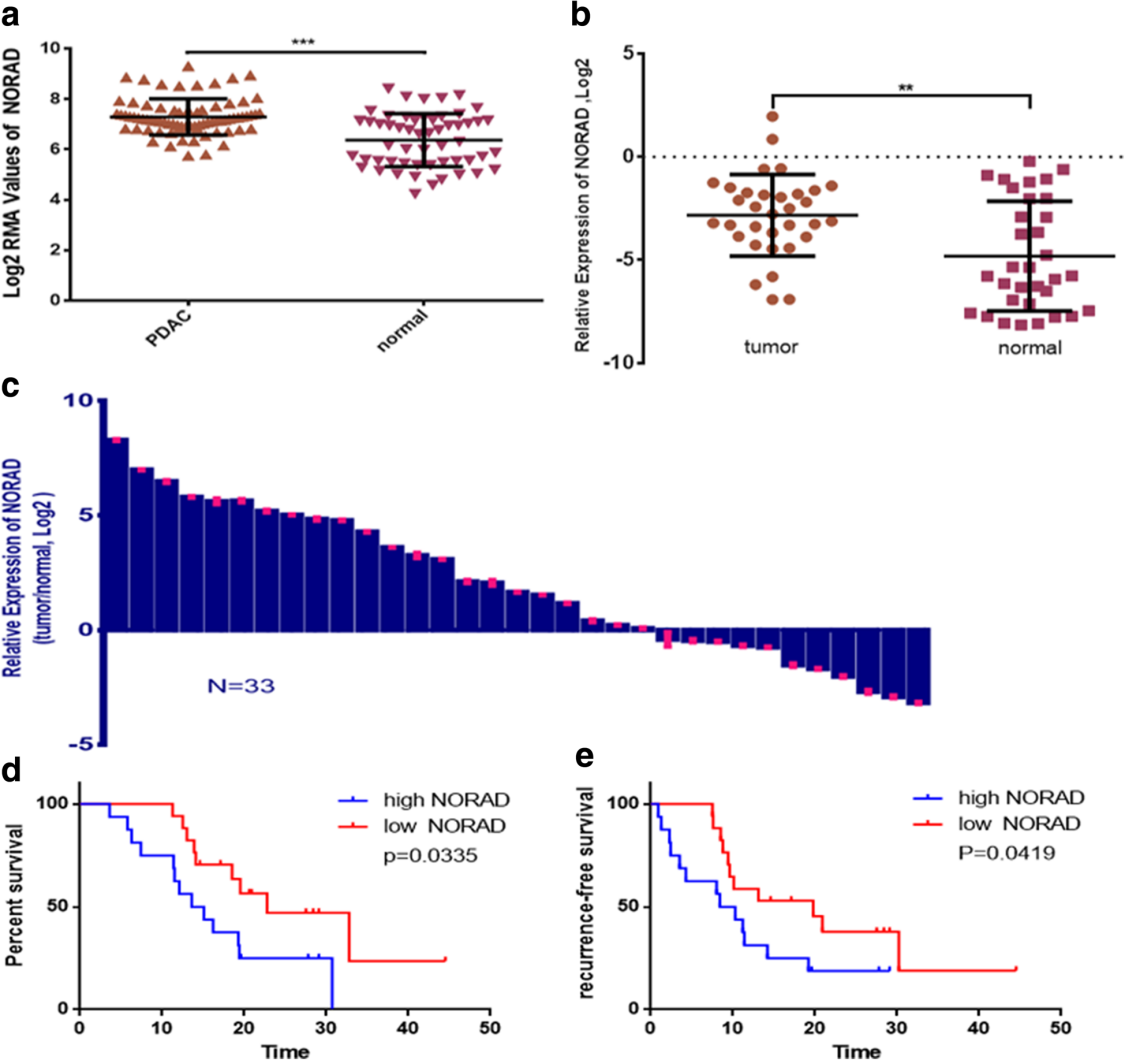
NORAD is upregulated in pancreatic cancer and is correlated with the prognosis of PDAC patients. **a** NORAD expression profiles in GSE15471 and GSE16515. The results are expressed as log2 RMA Values; **b** Relative NORAD expression in 33 paired PDAC as quantified by q-PCR. Results were expressed as log2 (2^-△△Ct^);**c** NORAD expression was investigated in the cancer tissue compared with its adjacent normal tissues. The results were presented as log 2-fold change of tumor tissues relative to normal tissues; **d**-**e** Kaplan–Meier estimator of overall survival (OS) and recurrence-free survival (RFS). *P* values were calculated by the log-rank test. *, statistical significance, *P* < 0.05, **p < 0.01, ***p < 0.001

The expression of NORAD was significantly higher in 75 pancreatic cancer samples than in 55 normal pancreas tissues (GSE15471 and GSE16515, *p* < 0.001, Fig. 6a). We then assayed NORAD expression in a panel of 33 PDAC with complete clinicopathological information (Additional file 5: Table S3), along with 33 paired normal pancreas tissues. NORAD was significantly upregulated in PDAC specimens compared with normal pancreas tissues (Fig. 6b, *p*<0.01). The expression of NORAD was also upregulated in most tumor tissues compared with the non-tumor tissues from the same donor (Fig. 6c), thus linking the upregulation of NORAD to pathological changes in pancreatic tissue.

Next, we evaluated the clinical significance of NORAD in PDAC. Thirty-three PDAC patients from our hospital were divided into two groups (high: *n* = 16, low: *n* = 17) according to the level of NORAD expression.

The Kaplan–Meier estimator was used to estimate recurrence-free survival (RFS) and overall survival (OS) rates and *p* values were calculated using the log-rank test. As shown in Fig. 6d-e, PDAC patients with higher NORAD expression have shorter overall survival (*p* = 0.0335) and recurrence-free survival (*p* = 0.0419) rates. The median overall and recurrence-free survival rates were sharply decreased (RFS: 9.35 versus 19.8 months; OS: 14.35 versus 22.8 months) in the high-NORAD group, which indicates that NORAD upregulation is associated with poor prognosis.

## Discussion

Pancreatic cancer is a devastating disease in which the majority of patients succumb within one year of diagnosis. The disease is characterized by a desmoplastic reaction that creates a dense microenvironment, promoting hypoxia and limiting cancer drug delivery due to decreased blood perfusion [7]. Stromal fibrosis and tissue necrosis are major histological sequelae of hypoxia [37]. Standard chemotherapeutic agents fail because they are unsuccessful at targeting cells within the hypoxic microenvironment. Out of all therapeutic obstacles, hypoxia might be the most important to target for elimination [38].

Responses to hypoxia are thought to operate via hypoxia-inducible factors (HIFs). Both hypoxia and overexpression of hypoxia-inducible factor (HIF)-1α and/or HIF-2α have been shown to promote EMT and metastatic phenotypes [39]. Mechanisms possibly connecting hypoxia with EMT include intracellular reactive oxygen species (ROS)-dependent HIF accumulation, Snail translocation and HIF1α-dependent accumulation of the transcription factors Snail, Twist, ZEB1 and ZEB2, key regulators of EMT [40]. Numerous studies have suggested that EMT both contributes to the early-stage dissemination of cancer cells in PDAC [5, 41] and induces chemoresistance, which may be more important to the unsatisfactory prognosis in pancreatic cancer [42].

Although various potential candidates emerge for disrupting EMT or treating advanced-stage pancreatic cancer [43–45], this cancer remains resistant to conventional therapies and is susceptible to early distant metastasis [46]. We believe that the mechanism of hypoxia-induced EMT is much more complicated than our current understanding and that the prognosis of pancreatic cancer will not be improved until we increase our understanding.

Recent evidence indicates that lncRNAs play essential roles in numerous biological processes, such as epigenetic regulation [47], cell cycle control [48], RNA decay and transcription [49, 50]. MiRNAs can function as master gene regulators, impacting a variety of cellular pathways involved in normal cellular functions as well as cancer development and progression [51]. LncRNAs that act as competing endogenous RNAs (ceRNAs) or natural microRNA sponges are important post-transcriptional regulators of gene expression that communicate with and co-regulate each other by competing for binding to shared microRNAs. Understanding this novel RNA crosstalk will lead to significant insights into gene regulatory networks and human development and disease [17].

NORAD is highly conserved and abundant, with expression levels of approximately 500–1000 copies per cell [21]. Notably, NORAD loss-of-function results in chromosomal instability and triggers dramatic aneuploidy in previously karyotypically stable cell lines. NORAD can directly regulate both ploidy and chromosomal stability by sequestering PUMILIO proteins, which repress key mitotic, DNA repair, and DNA replication factors [52]. NORAD is a cytoplasmic multivalent PUMILIO binding platform that acts as a negative regulator of PUMILIO activity [21]. In breast cancer, NORAD knockout significantly suppressed tumor cell growth and proliferation, suggesting that it plays an oncogenic role [22]. However, this study was unable to shed light on its mechanism.

Previous studies have revealed that miR-125a-3p plays a tumor suppressive role in many cancers, including non-small-cell lung cancer [53], gastric cancer [54], hepatocellular carcinoma [55] and myeloma [56]. Moreover, miR-125a-3p is widely involved in cell proliferation [53], apoptosis [57], differentiation, as well as migration and invasion. However, little is known about its function in PDAC or its link to NORAD. Only one study reports that assessing the levels of miR-125a-3p in combination with 7 other miRNAs can be clinically valuable for identifying patients with pancreatic and biliary-tract cancers who could benefit from surgical intervention [58].

**Fig. 7:**
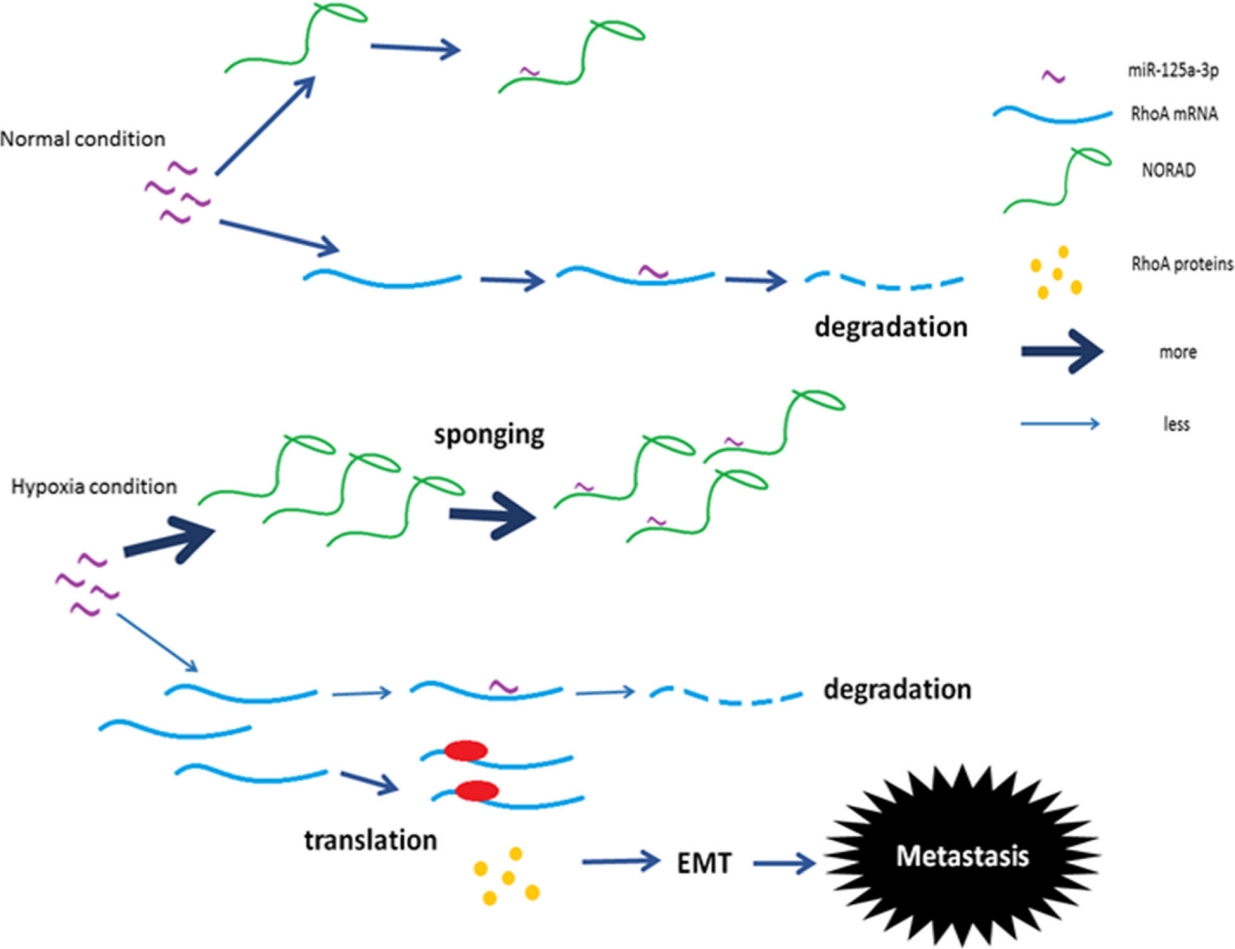
Schematic diagram showing that NORAD increases the expression of RhoA proteins via sponging of its inhibitor miR-125a-3p, thus bridging hypoxia and metastasis

To the best of our knowledge, we are the first to demonstrate that NORAD acts as a ceRNA of miR-125a-3p and that it is involved in regulating pancreatic cancer metastasis and invasion. In addition, miR-125a-3p has also been reported to participate in several other pathways such as the p53 pathway [59], the p38/ MAPK pathway [60] and the PI3K/AKT/mTOR pathway [55], all of which may be associated with the malignant phenotype mediated by NORAD/miR-125a-3p. In the present study, we focused on RhoA, which is not only an important downstream factor in hypoxia but also a crucial regulator of EMT. According to bioinformatics analyses, RhoA and NORAD share the same response elements for miR-125a-3p. We further confirmed that the expression level of miR-125a-3p can be downregulated by NORAD and that miR-125a-3p can bind to NORAD directly at its MRE. We found that NORAD-wt increased RhoA, NORAD knockdown downregulated RhoA, and NORAD-mut failed to increase RhoA levels. Knock-down of NORAD could partially rescue the hypoxia-induced suppression of RhoA and EMT. Thus, NORAD may act as a ceRNA to regulate the expression of RhoA by sequestering its inhibitor miR-125a-3p, thus bridging the response to hypoxia and EMT (Fig. 7).

## Conclusion

In conclusion, we determined that NORAD is an independent prognostic factor for PDAC patients and that it promotes the metastasis and invasion of PDAC both in vitro and in vivo. We also shed light on the role of NORAD in hypoxia-induced EMT. We found that hypoxia can increase NORAD expression and stimulate EMT in pancreatic cancer cells and that NORAD promotes EMT and metastasis by regulating RhoA in a miR-125a-3p-dependent manner. Knockdown of NORAD can alleviate malignant phenomena caused by hypoxia. Thus, we believe that NORAD may be a potential novel therapeutic target for the treatment of PDAC.

## Additional files

Additional file 1: Box plots of GSE15471 and GSE16515. (TIFF 154 kb)

Additional file 2: Primers used for qRT-PCR assays. (XLSX 9 kb)

Additional file 3: NORAD expression among 8 cell lines. (TIFF 728 kb)

Additional file 4: The expression of RhoA and miR-125a-3p in GSE32688. (XLSX 11 kb)

Additional file 5: Clinicopathological information of 33 PDAC. (XLSX 9 kb)
