# Supplementary figures and images for: Long noncoding RNA NORAD, a novel competing endogenous RNA, enhances the hypoxia-induced epithelial-mesenchymal transition to promote metastasis in pancreatic cancer
Source: Mol Cancer. 2017 Nov 9;16:169. doi: 10.1186/s12943-017-0738-0 (PMC5679488; doi:10.1186/s12943-017-0738-0)

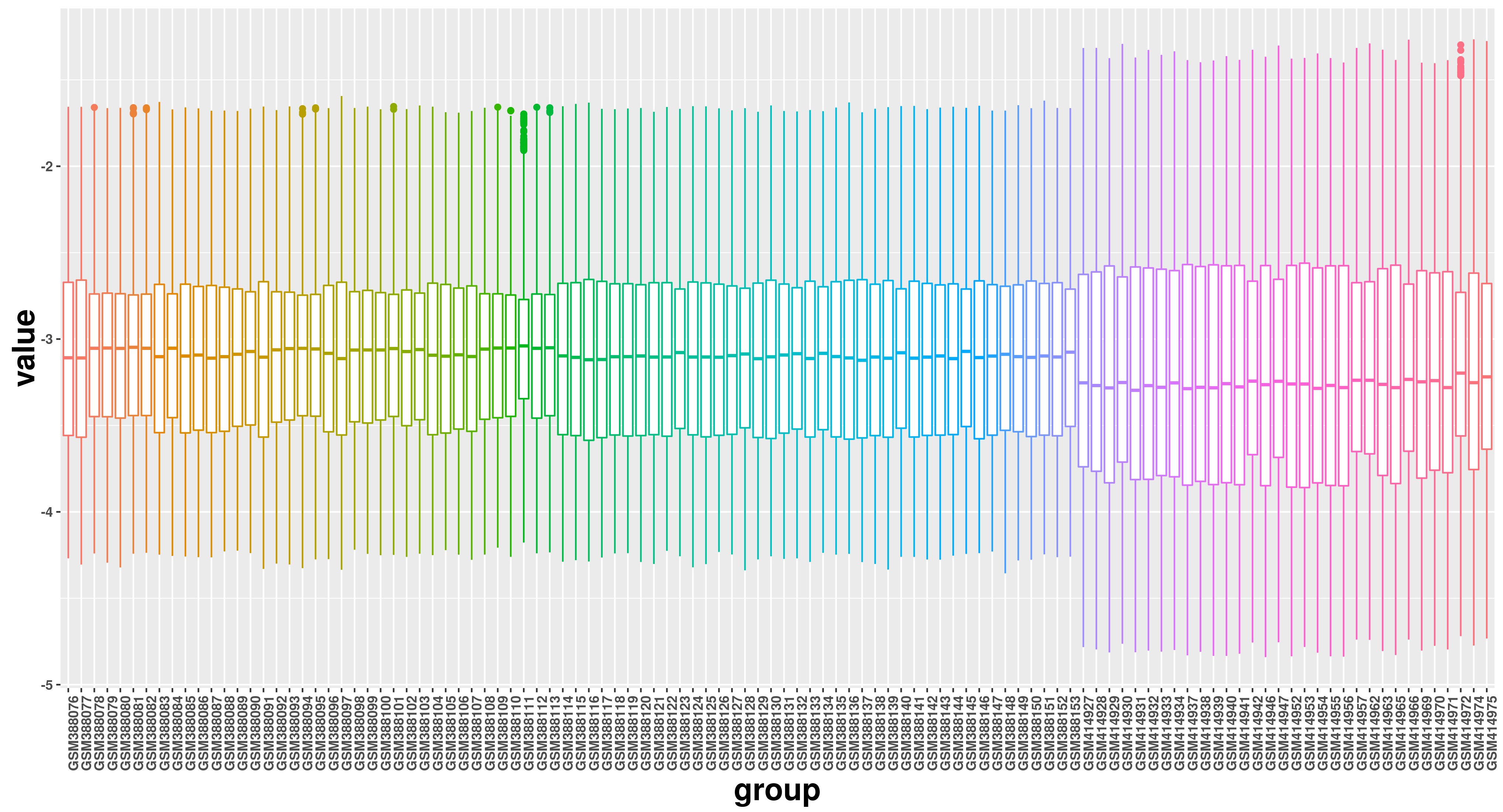

Supplement: Supplementary file 1 — Box plots of GSE15471 and GSE16515. (TIFF 154 kb) [file 12943_2017_738_MOESM1_ESM.tif]

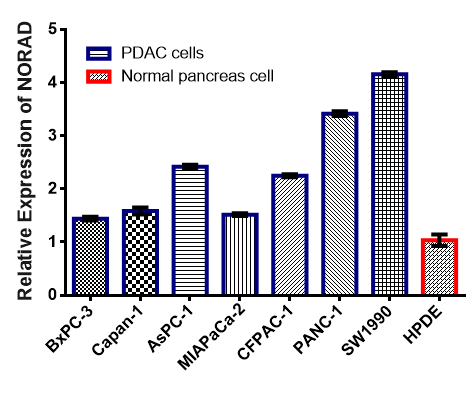

Supplement: Supplementary file 3 — NORAD expression among 8 cell lines. (TIFF 728 kb) [file 12943_2017_738_MOESM3_ESM.tif]
